# Bioorthogonal mRNA labeling at the poly(A) tail for imaging localization and dynamics in live zebrafish embryos†
†Electronic supplementary information (ESI) available. See DOI: 10.1039/c9sc05981d


**DOI:** 10.1039/c9sc05981d

**Published:** 2020-02-24

**Authors:** Kim J. Westerich, Karthik S. Chandrasekaran, Theresa Gross-Thebing, Nadine Kueck, Erez Raz, Andrea Rentmeister

**Affiliations:** a Cells in Motion Interfaculty Centre (CiMIC) , Waldeyerstraße 15 , D-48149 Münster , Germany; b Institute of Cell Biology Center for Molecular Biology of Inflammation , University of Münster , D-48149 Münster , Germany . Email: erezraz@uni-muenster.de; c Institut für Biochemie , Westfälische Wilhelms-Universität Münster , Wilhelm-Klemm-Str. 2 , 48149 Münster , Germany . Email: a.rentmeister@uni-muenster.de

## Abstract

Live imaging of mRNA in cells and organisms is important for understanding the dynamic aspects underlying its function. Ideally, labeling of mRNA should not alter its structure or function, nor affect the biological system. However, most methods applied *in vivo* make use of genetically encoded tags and reporters that significantly enhance the size of the mRNA of interest. Alternately, we utilize the 3′ poly(A) tail as a non-coding repetitive hallmark to covalently label mRNAs *via* bioorthogonal chemistry with different fluorophores from a wide range of spectra without significantly changing the size. We demonstrate that the labeled mRNAs can be visualized in cells and zebrafish embryos, and that they are efficiently translated. Importantly, the labeled mRNAs acquired the proper subcellular localization in developing zebrafish embryos and their dynamics could be tracked *in vivo*.

## Abstract 2

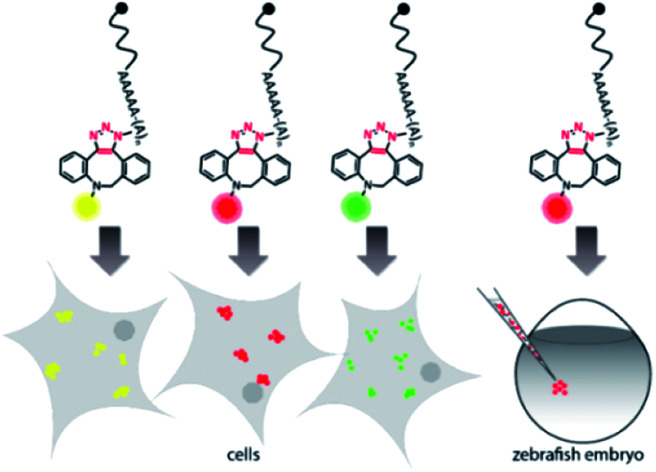
Live imaging of mRNA in cells and organisms is important for understanding the dynamic aspects underlying its function.

## Introduction

The defined localization of different mRNAs to specific subcellular domains provides a mechanism for regulating gene expression temporally and spatially, in particular during dynamic processes such as embryonic development.^1^–^7^ Classical examples of localized mRNAs include the *ASH1* in yeast,^8^ the *bicoid*, *oskar* and *nanos* in *Drosophila* embryos,^9^ the *vg1* in *Xenopus* oocytes,^10^ as well as *beta actin* in mammalian neurons.^11^–^13^ The development of the germline is a good model for studying subcellular mRNA localization, as it heavily relies on tight post-transcriptional regulation, with many key components being highly conserved across species. This includes the asymmetric localization of *vasa* and *nanos* (*nos*) mRNA in primordial germ cells, the progenitors of the germline lineage that gives rise to egg and sperm (PGCs).^5^,^14^–^27^ Such key processes are studied in zebrafish by typically employing genetically encoded tagged mRNAs and reporters that require significant changes at the sequence level of the mRNA of interest.^28^

Various approaches have been developed to visualize the subcellular localization of mRNAs in living systems and to gain a more comprehensive understanding of the life-cycle of an mRNA.^29^–^32^ Among those, genetically encoded fluorescent proteins fused to RNA-binding proteins have dominated RNA-imaging in living organisms till now. The widely used MS2-GFP system requires tagging of the target-mRNA molecules with 24 MS2 hairpin sequences at the 3′ untranslated region (UTR) (resulting in 48 bound GFP molecules) to enable single-molecule RNA detection in live mammalian cells.^33^ The large size of the tag can cause tag-aggregate formation,^34^ thus affecting the trafficking behavior of the RNA of interest^35^ and therefore approaches based on much smaller fluorogenic aptamer tags were developed.^36^,^37^ Alternatively, direct and tag-independent RNA labeling can be achieved by a RCas9-GFP fusion.^38^ While this enabled native mRNA-tracking, this strategy still requires recruiting a relatively large exogenous protein (RCas9) to the RNA of interest.

Visualization of mRNA can also be achieved by utilizing fluorescently labeled probes for *in situ* hybridization. Examples include single-molecule fluorescence *in situ* hybridization (smFISH),^39^ RNAScope,^40^ FIT probes,^41^ molecular beacons,^42^ sequence-encoded amplicon (SeqEA)^43^ and multiplexed error-robust fluorescence *in situ* hybridization (MERFISH).^44^ These approaches are typically applied in fixed cells and enable single-molecule detection and resolution.^45^ Out of these, FIT probes and molecular beacons lead to increased fluorescence emission upon target RNA binding and can thus be used in living cells and organisms where excess probe cannot be simply washed away.^46^–^48^

To circumvent the introduction of exogenous probes like those mentioned above, several approaches for direct covalent labeling of mRNA with small fluorophores were devised. Body labeling by statistical incorporation of modified nucleotides during transcription and subsequent conjugation chemistry allows for multiple labeling.^49^–^51^ Recently, the modified nucleoside ethynyl uridine (EU) was used to label nascent transcripts, thereby highlighting transcription of RNA in the brains of developing zebrafish larvae. Currently, this is the only study where click chemistry is used for labeling RNAs in this model.^52^ This approach is however limited in that it does not allow following specific mRNA molecules. Other studies utilized EU or azido-functionalized uridine to image RNA in fixed cells^53^ and various tissues from mice.^54^ However, the presence of the modified nucleotides throughout the sequence of the RNA could potentially affect the translational activity and the structure of the molecule.

Alternatively, by adding a suitable genetically encoded tag in the UTR, RNAs can be labeled in cells using RNA-modifying enzymes recognizing this structural element.^55^–^61^ We showed previously that the hallmarks of eukaryotic mRNA – namely the 5′-cap and the poly(A) tail – can be site-specifically labeled using chemo-enzymatic approaches.^62^–^65^ Both labeling approaches rely on small fluorescent dyes and do not alter the coding sequence or the UTR, and therefore do not interfere with coding or regulatory elements of the mRNA. However, the cap labeling approach was limited regarding the number of fluorophores that could be attached and caused interference with translation-initiation.^66^–^68^ The poly(A) tail labeling allowed introduction of multiple fluorophores per RNA molecule and did not inhibit translation, making it a valid strategy for RNA visualization in cells and suggesting it could be even utilized *in vivo*.^62^

## Experimental section

Experimental details are given in the ESI Section.†

## Results and discussion

**Fig. 1:**
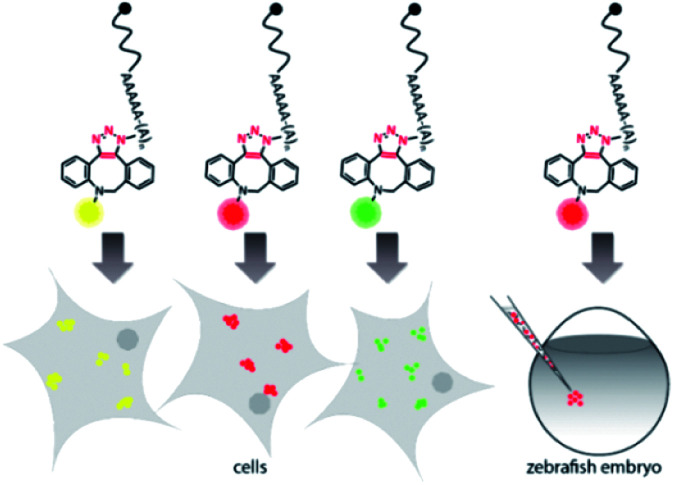
Concept of visualizing bioorthogonally labeled mRNAs in mammalian cells (left) or zebrafish embryos.^15^ Reporter-mRNAs were labeled at the 3′ poly(A) tail using azido-ATP and strain-promoted azide–alkyne cycloaddition with DBCO-fluorophores.

Harnessing the potential of this labeling strategy, in the current study we investigated if different types of fluorophores could be used to visualize mRNA in living systems. We therefore produced capped reporter mRNAs by *in vitro* transcription and then incorporated azido-modified adenosine nucleotides at their poly(A) tails using the yeast poly(A) polymerase (yPAP) and 2′-azido-2′-dATP (as co-substrate) as previously described.^51^,^62^ Then, a set of eight different dibenzocyclooctyne (DBCO)-conjugated fluorophores covering different emission wavelengths was tested for labeling azido-modified *egfp* and *mcherry* mRNAs in a strain-promoted alkyne–azide cycloaddition (SPAAC) (Fig. 1), namely – AF488, carboxyrhodamine 110 (CR110), AF555, AF647, sulfo-Cy5 (Cy5), sulforhodamine B (SRB), TAMRA and Texas Red (Fig. S1†).

**Fig. 2:**
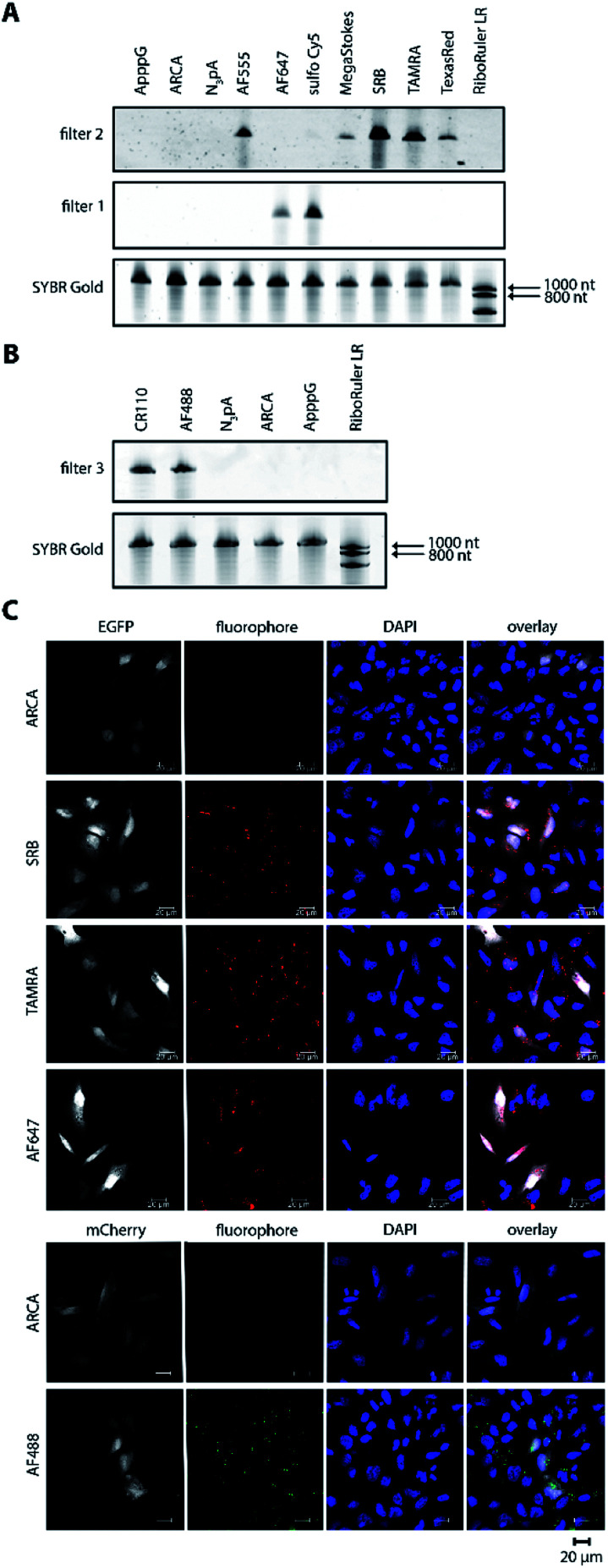
Bioorthogonal labeling of mRNAs at the poly(A) tail with different fluorophores enables their visualization in HeLa cells. (A) PAGE analysis of *egfp* mRNAs labeled with indicated fluorophores or controls (ApppG, ARCA, N_3_pA). (B) PAGE analysis of *mcherry* mRNAs labeled with indicated DBCO-fluorophores or controls. (7.5% PA gel, 20 W, 2.5 h, rt; filter 1: ≥575 nm, filter 2: ≥665 nm, filter 3: ≥510 nm). (C) Confocal microscopy of HeLa cells transfected with *egfp* or *mcherry* mRNA with indicated labels. Signals (red or green dots) from the fluorophores, EGFP/mCherry and DAPI channels enabled visualization of mRNA, EGFP/mCherry and nuclei, respectively. Scale bar = 20 μm.

For the 5′-cap, the anti-reverse cap analog (ARCA, 3′-*O*-Me-m^7^G(5′)ppp(5′)G) resulting in correct cap orientation and translation was used in all cases, except for the negative control, where an ApppG-capped mRNA was used to rule out cap-independent translation. As an additional control, the non-labeled ARCA-capped mRNA with azido-modifications (N_3_pA) at the 3′ poly(A) tail was produced. Analysis of the labeled mRNAs showed that fluorescent signals at the respective wavelength settings could be observed in all cases for ∼1200 nt RNAs (Fig. 2A, B and Table S1†). Unlabeled controls (ApppG, ARCA, N_3_pA) did not show fluorescent signals. Together with SYBR Gold staining (confirming the presence of all mRNA samples) these data demonstrate that the mRNAs remain intact and are efficiently labeled by various DBCO-fluorophore conjugates (Fig. 2A, B and S8†).

Next, we tested whether the differently labeled mRNAs can be visualized in cells. To this end, we transfected HeLa cells with each of the labeled mRNAs and imaged them using confocal microscopy (Fig. 2C, spectra and filter settings in Table S2†). We found that mRNAs labeled with either SRB, TAMRA, AF647 or AF488 could be detected as fluorescent dots distributed in perinuclear and cytosolic regions (red or green dots in Fig. 2C), similar to previous findings regarding mRNAs labeled by different methods. Furthermore, mRNAs labeled with AF555, Texas Red, sulfo-Cy5 (Cy5) or CR110 gave similar signals after transfection of HeLa cells (Fig. S2 and S3†). These results indicate that the fluorophores are tolerated by HeLa cells irrespective of their structural make-up.

To confirm that the labeling of mRNAs with different fluorophores did not interfere with downstream processing, *i.e.* translation, the resulting EGFP- and mCherry-protein levels were assessed in whole cell extracts from HeLa cells transfected with the same set of labeled and unlabeled transcripts as the cell imaging experiments (Fig. S4 and S5†). Indeed, *egfp* and *mcherry* mRNAs labeled with the different DBCO-fluorophores were optimally translated, showing high levels of EGFP and mCherry expression, similar or even higher that of ARCA- and N_3_pA-mRNAs. The negative control (ApppG-mRNA) was not translated, ruling out cap-independent translation. Together, these results show that bioorthogonal labeling of mRNA at the poly(A) tail *via* the SPAAC reaction can be used to incorporate different types of fluorescent dyes. Importantly, the differently labeled mRNAs can be visualized in cells and are actively translated by the cellular machinery.

Next, we examined whether this labeling approach would be compatible with studying mRNAs *in vivo*. We used zebrafish embryos as a model, since they can be injected with mRNAs at the 1-cell stage, with the RNAs distributed to all other cells of the embryo during subsequent cell divisions and development. Furthermore, the unique optical properties of the embryos allow imaging fluorescent signals resulting from injection of labeled mRNAs. We fused a reporter mRNA to the 3′ UTR of *nanos* (*nos* 3′ UTR), since this *cis*-acting element leads to protection and translation of the transcript in PGCs of zebrafish embryos,^69^ in contrast to the fate of the mRNA in other cell types in which *nos* 3′ UTR-containing mRNAs are degraded and translationally suppressed by microRNAs.^18^,^20^

**Fig. 3:**
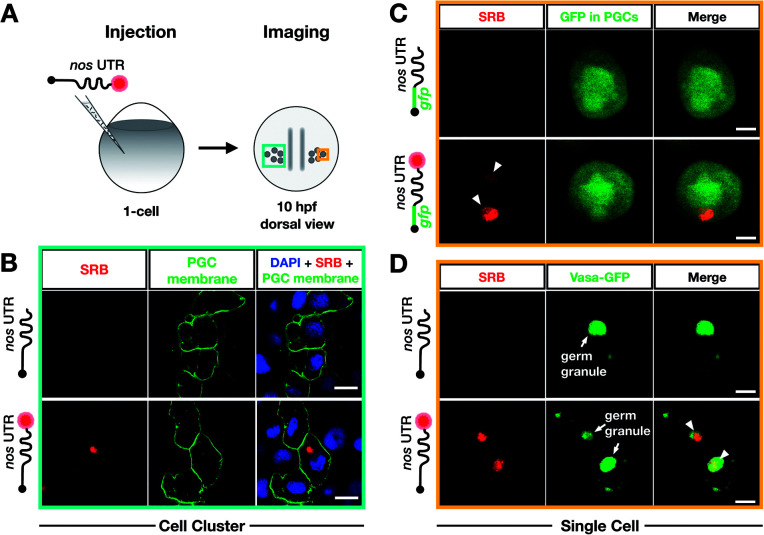
Bioorthogonal labeling with DBCO-SRB reveals subcellular localization of mRNA injected into zebrafish embryos without interfering with transcript positioning and translation. (A) 1-cell stage zebrafish embryos were injected with mRNA containing the PGC-specific *nos* 3′ UTR synthesized *in vitro*, which was either not labeled or labeled with SRB (red circle). PGCs were imaged 10 hpf, focusing either on a cell cluster (as in B) or on single cells (as in C and D). (B) Injection of SRB-labeled-mRNA containing a non-translatable *mcherry* sequence upstream of *nos* 3′ UTR (STOP *mcherry-nos*) results in specific detection of the transcript in cells expressing the transgenic PGC membrane marker *egfp-f′ nos* (lower panels), while non-labeled mRNA is not visible (upper panels). An overlay of the two detection channels and DAPI (labeling the nuclei) is presented in the right panels. Scale bar = 10 μm. (C) Injection of *gfp-nos* mRNA results in GFP-expression in PGCs (as in ref. 69, upper panels). SRB-labeling of the mRNA does not interfere with PGC-specific GFP-expression and results in detection of granule-like mRNA structures (arrowheads, lower panels). An overlay of the detection channels is presented in the right panels. Scale bar = 5 μm. (D) Co-injection of SRB-labeled STOP *mcherry-nos* mRNA with mRNA encoding for the germ granule marker protein Vasa GFP (arrows, Fig. S7†), reveals the localization of labeled STOP *mcherry-nos* transcripts to germ granules in PGCs (arrowheads, lower panels). Non-labeled mRNA is not visible (upper panels). An overlay of the detection channels is presented in the right panels. Scale bar = 5 μm.

**Fig. 4:**
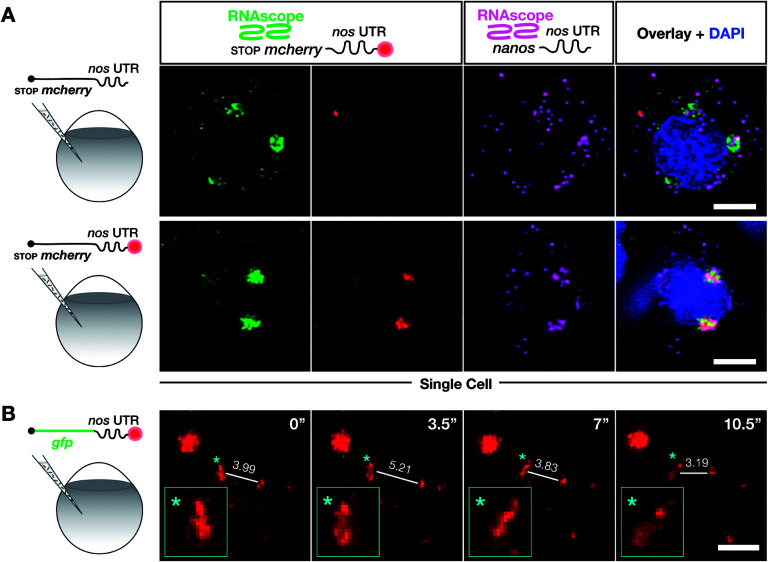
Labeling with DBCO-SRB does not alter the subcellular localization of injected mRNA and allows resolving the dynamic positioning of the transcript. (A) 1-cell stage embryos were injected with *in vitro* synthesized mRNA containing a non-translatable *mcherry* sequence upstream of *nos* 3′ UTR (STOP *mcherry-nos* mRNA). The injected mRNA was either not labeled (upper panels) or labeled (lower panels) with SRB (emission 610 nm). After 10 hpf, the embryos were fixed and hybridized with RNAscope probes,^71^ targeting the *mcherry* sequence (labeling the injected mRNA, emission filter 509 nm) and that of *nanos* open reading frame (labeling the endogenous *nanos* mRNA, emission filter 647 nm). An overlay of the three detection channels and DAPI (labeling the nuclei, emission filter 470) is presented in the right panels. (B) SRB-labeled *gfp-nos* mRNA was injected into 1-cell stage zebrafish embryos and tracked over time in PGCs after 1 day of development. The panels show maximum intensity projections spanning 9 μm, with time intervals of 3.5 seconds between frames. Lines highlight changes in distance (μm) between the edges of two RNA-containing structures over time. Changes in the shape of an RNA-containing structure (marked by an asterisk) are presented in the insets on the bottom left of each plane. Scale bar = 5 μm.

To test whether poly(A) tail-labeling of mRNAs containing a *nos* 3′ UTR would be suitable for visualization in zebrafish embryos, we injected the *in vitro*-labeled mRNA into 1-cell stage embryos and assessed the dye signal in PGCs at 10 hours post fertilization (hpf) (Fig. 3A). For this purpose, two different mRNAs were constructed (Fig. S7†). First, mRNA containing a non-translatable *mcherry* sequence fused to the *nos* 3′ UTR (STOP *mcherry-nos*) was generated. This mRNA served for co-visualization of the injected labeled RNA with PGC-specific fluorescent markers (Fig. 3B and D), and allowed the use of a specific probe targeting the *mcherry* sequence in a subsequent *in situ* hybridization analysis, thereby distinguishing between labeled and endogenous *nos* 3′ UTR-containing mRNAs (Fig. 4A). Second, mRNA containing a *gfp* sequence fused to the *nos* 3′ UTR (*gfp-nos*) was constructed to assess whether PGC-specific translation of the labeled transcript can be achieved (Fig. 3B). We chose SRB to label mRNAs (Fig. S6†) as it provided reliable and consistent signals as compared to other fluorophores (data not shown).

We first injected DBCO-SRB-labeled STOP *mcherry-nos* mRNA into 1-cell stage transgenic zebrafish embryos engineered to express EGFP on the membrane of PGCs and assessed the signal of the SRB-labeled RNA (Fig. 3B). Importantly, the labeled nos 3′ UTR-containing mRNA was specifically found within the EGFP-labeled PGCs (Fig. 3B, lower panels), in line with previous findings concerning this type of mRNA when non-labeled.^69^ As expected, no fluorescent signal was detectable in the 610 nm emission channel in embryos injected with non-labeled mRNA (Fig. 3B, upper panels). These data indicate that labeling mRNAs at the poly(A) tail with small fluorescent dyes is suitable for visualizing their localization in zebrafish embryos, and does not interfere with the nos 3′ UTR-mediated mRNA stabilization within the PGCs or degradation in other cell types.

Next, we set out to investigate whether poly(A) tail-labeled mRNA remains functional with respect to translation in zebrafish embryos. To this end, *gfp-nos* mRNA was injected into 1-cell stage zebrafish embryos, which were subsequently assessed for GFP signal at 10 hpf (Fig. 3C). We found that both non-labeled (Fig. 3C, upper panels) and SRB-labeled (Fig. 3C, lower panels) *gfp-nos* mRNAs expressed GFP specifically in PGCs. These data show that the translation of the mRNA is not affected by the label.

In the next step, we tested whether the labeled mRNA could be accurately localized within the PGCs. As *nanos* mRNA is known to be localized to germ granules (non-membrane-bound granules that contain RNAs and proteins important for germline development) within PGCs,^70^ we tested whether the SRB-label would affect this subcellular localization of the injected mRNA. This experiment was conducted by co-injecting the STOP *mcherry-nos* mRNA described above with the mRNA coding for the germ granule marker-protein Vasa-GFP and by assessing their subcellular localization within PGCs at 10 hpf (Fig. 3D). Indeed, the SRB-label reveals a distinct subcellular pattern of the mRNA that overlaps with the position of the germ granule marker Vasa (Fig. 3D, lower panels).

To further verify that SRB-labeling does not affect the fate and localization of the injected mRNA, we then compared the subcellular localization of injected non-labeled and SRB-labeled mRNAs with the location of endogenous *nanos* mRNA using the highly sensitive *in situ* hybridization method RNAscope, which is conducted on fixed embryos.^71^

We found that the non-labeled STOP *mcherry-nos* mRNA (Fig. 4A upper left panel, with the *mcherry* sequence targeted by an RNAscope probe) was localized to granules within which the endogenous *nanos* mRNA resides (Fig. 4A upper third panel, with the *nanos* open reading frame targeted by an RNAscope probe). Furthermore, when the same experiment was performed with labeled STOP *mcherry-nos* mRNA, the SRB signals co-localized with the RNAscope signals for injected and endogenous *nos* mRNA (Fig. 4A lower panels). Last, no non-specific signal, potentially caused by putative SRB-detachment or degradation of the mRNA, is detected. These data show that the subcellular localization of injected mRNA (that was produced and labeled *in vitro*) resembles that of endogenous *nanos* mRNA, which is deposited in the egg during oogenesis in the mother and is associated with different mRNA-binding proteins that control its localization and function in PGCs—an important feature for application in biological studies. Importantly, the SRB-label does not significantly affect the behavior of the transcript *in vivo*.

We next wanted to test whether poly(A) tail-labeling of mRNAs would allow us to track the dynamic positioning of transcripts *in vivo*. This would enable studying the dynamics of subcellular mRNA localization in the living organism, which is important for understanding the extensive regulation of mRNA localization and fate from transcription to translation.^72^ To this end, we injected the SRB-labeled *gfp-nos* mRNA into 1-cell stage zebrafish embryos. Time-lapse analysis of the SRB-signal in PGCs of 1 day-old embryos revealed dynamic changes in the structure and subcellular localization of the labeled mRNA (Fig. 4B, Movie S1†), that could allow one to follow and analyze different processes of RNA localization and transport. The bioorthogonally attached label can thus serve as a real-time reporter of mRNA localization in live cells in the context of the developing embryo.

**Fig. 5:**
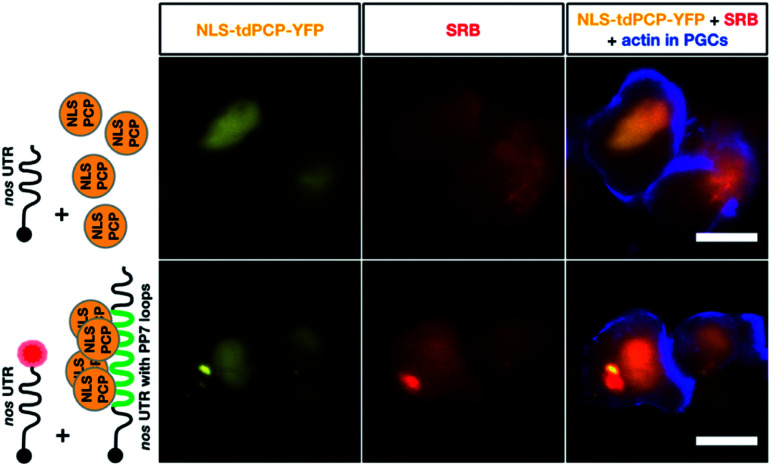
Co-detection of *nos* 3′ UTR-containing mRNA employing two different RNA labeling systems for live imaging *in vivo*. The same transcript type was visualized using click chemistry-based labeling and the PP7 detection system. To visualize *nos* 3′ UTR-containing mRNA using click chemistry, 1-cell stage embryos were injected with *in vitro* synthesized STOP *mcherry-nos* mRNA, which was either not labeled (upper middle panel) or labeled (lower middle panel) with SRB (red circle). To simultaneously detect *nos* 3′ UTR-containing mRNA using the PP7 detection system, *in vitro* synthesized mRNA encoding for the PP7 coat protein (NLS tdPCP-YFP) was injected either alone (upper left panel) or together with *nos* 3′ UTR-containing mRNA that bears a sequence of 24 loops in its 3′ UTR region, to which multiple copies of the coat protein can bind (*nanos-nos* 24xPP7 mRNA, lower left panel). PGCs were visualized by additional injection of a PGC-specific mRNA encoding for the actin marker protein Lifeact-tagBFP-nos. Scale bar = 10 μm.

Finally, we compared the click chemistry-based RNA labeling approach with the previously established PP7 detection system. For this purpose, zebrafish embryos were injected with mRNA constructs allowing co-detection of the *nos* 3′ UTR-containing transcript by both systems in the same cell (see Fig. 5). As presented above, we utilized *in vitro*-labeled *nos* 3′ UTR-containing mRNA (STOP *mcherry-nos*) to visualize the transcript employing the click chemistry approach.^28^,^73^ For RNA detection employing the PP7 system, we designed a second *nos* 3′ UTR-containing mRNA that bears loop structures recognized by a YFP-tagged PP7 coat protein (PCP, see methods,^73^). The signals corresponding to each of the labeled mRNAs were assessed in PGCs at 10-11 hpf. Importantly, both the click-attached SRB dye and the YFP-labeled PCP protein allowed the visualization of *nos* 3′ UTR-containing mRNA in live PGCs (lower panels in Fig. 5). Both detection systems reveal the characteristic granular arrangement of *nanos* mRNA in the germ cell, with the PCP protein labeling the nucleus as well.

According to these data, the click chemistry labeling approach and the PP7-based system provide comparable information concerning the localization of the mRNA. Nevertheless, the two systems could show differences in certain cases concerning localization and dynamics of the RNA. Such differences could result from the fact that labeling RNAs by click chemistry does not involve alteration of the mRNA sequence, while the PP7-based detection involves large RNA sequence insertions (over 1400 bases). The added RNA sequence could potentially affect different characteristics of the tracked RNA, as it is relatively long (*e.g.* the *nos* 3′ UTR itself is only 631 bp long). In addition to the extended length of the RNA, unlike the click-based labeling, the PP7 system requires tethering 48 fluorescently-tagged proteins to the RNA, which could potentially further affect the dynamics of the labeled RNA.

## Conclusions

In summary, we demonstrate a generally applicable bioorthogonal approach to label mRNAs in a way that maintains their translational activity in adherent cells and zebrafish embryos, and does not affect their subcellular localization in the latter. We show that an mRNA can be efficiently labeled at its 3′ poly(A) tail with different fluorophores, and that the label facilitates mRNA-visualization in cells and in developing embryos. Importantly, the labeled mRNA could be imaged and tracked in real-time in a living vertebrate, as demonstrated by experiments with mRNAs containing localization-specifying 3′ UTRs. Since mRNAs can be labeled with fluorophores of different spectra, this strategy is expected to pave the way for labeling and imaging more than one mRNA, enabling the correlation between localizations and functions of the mRNAs of interest. Furthermore, the procedure we present involves very small alterations of the mRNA, especially when compared to current methods such as the MS2 or PP7 system. Specifically, the stem-loops, coat-protein and fluorescent tag (GFP-MCP) in the MS2 system together are in the order of 25 000–50 000 g mol^–1^, whereas the fluorophores employed in the current work weigh only 500–2500 g mol^–1^. Thus, the poly(A) tail labeling technique we describe facilitates labeling mRNA with minimal effects or interference with its functions (translation and localization), and enables live-imaging. These methods are expected to aid in understanding the regulation of mRNA function in diverse processes in cells, embryonic development and disease contexts.

## Conflicts of interest

There are no conflicts to declare.

## Supplementary Material

Supplementary information

Supplementary movie
