# Supplementary material for: Bioorthogonal mRNA labeling at the poly(A) tail for imaging localization and dynamics in live zebrafish embryos
Source: Chem Sci. 2020 Feb 24;11(11):3089–95. doi: 10.1039/c9sc05981d (PMC7879197; doi:10.1039/c9sc05981d)
Supplement: Supplementary file 1 [file SC-011-C9SC05981D-s001.pdf]

Electronic Supplementary Material (ESI) for Chemical Science.
This journal is © The Royal Society of Chemistry 2020

Bioorthogonal mRNA labeling at the poly(A) tail for imaging localization and dynamics in live zebrafish embryos

Kim J. Westerich,‡,[b] Karthik S. Chandrasekaran,‡,[c] Theresa Gross-Thebing,[b] Nadine Kück,[c] Erez Raz*[a,b] and Andrea Rentmeister*[a,c]

Table of Contents

Table of Contents 2
Experimental Procedures 2
Enzymatic synthesis of mRNAs modified at the 5' cap and the 3' poly(A) tail 2
Assessment of efficiency of SPAAC reaction 2
Cell culture and confocal imaging 2
Cell culture and western blot analysis 3
Zebrafish work 3
Construction of plasmids, mRNA synthesis and labeling 3
Microinjection into zebrafish embryos 3
RNAscope assay 4
Microscopy and image analysis in zebrafish embryos 4
Supplementary figures 5
Supplementary tables 12
References 12
Author Contributions 12

Experimental Procedures

Enzymatic synthesis of mRNAs modified at the 5' cap and the 3' poly(A) tail

The *egfp* and *mcherry* mRNAs were synthesized using the eGFP pMRNAxp mRNAExpress™ and mCherry pMRNAxp mRNAExpress™ vectors as templates.[1] First, the DNA template was amplified in 1 × HF puffer using plasmid (70 ng), dNTP mix (0.5 mM), forward primer (5'-TAA TAC GAC TCA CTA TAG GGA AAT AAG-3', 0.5 µM), reverse primer (5'-GTC GAC ACT AGT TCT AGA GCG-3', 0.5 µM) and Phusion High-Fidelity DNA Polymerase (1 U). The resulting DNA was used as template (100 ng) for *in vitro* T7 transcription performed in 1 × transcription buffer by adding A/C/UTP mix (0.5 mM), GTP (0.1 mM), ApppG or ARCA cap analog (1 mM), RiboLock RNase Inhibitor (30 U), T7 RNA polymerase (50 U) and pyrophosphatase (0.1 U) for 3 h at 37°C. Remaining DNA template was digested by incubation with 2 U of DNase I for 1 h at 37°C and then, mRNAs were purified using the RNA Clean & Concentrator™-5 Kit (*Zymo Research*). The ARCA-capped *egfp* or *mcherry* mRNA (4000 ng) alone was subjected to enzymatic addition of 2'-N_3-2'-dATP (1 mM; TriLink Biotechnologies) to the poly(A) tail in the presence of yeast poly(A) polymerase (600 U) in 1 × PAP reaction buffer for 1 h at 37°C followed by isopropanol precipitation overnight and washing with 70% ethanol. The click reactions were performed with azido-modified mRNA (1000 ng) and DBCO-conjugated AF555 or AF647 or SRB or TAMRA or TexasRed or AF488 or Sulfo-Cy5 or CR110 or alkyne-MegaStokes (75 µM; Jena Biosciences) for 1 h at 37°C followed by isopropanol precipitation overnight and washing with 70% ethanol. Then, the modified and labeled mRNAs were analyzed by 7.5% denaturing PAGE and imaged using the Typhoon FLA 9500 scanner and settings tabulated in Table S1. The Phusion PCR primers were from Biolegio. Phusion High-Fidelity DNA Polymerase, RiboLock RNA Inhibitor, T7 RNA polymerase, pyrophosphatase, DNase I and Yeast Poly(A) Polymerase (PAP) were purchased from Thermo Fisher Scientific. The $m_2^{7,3'-O}GP_3G$ (ARCA) and the G(5')ppp(5')A (ApppG) cap analogs were purchased from Jena Bioscience. RiboRuler Low Range RNA Ladder for denaturing PAGE was from Thermo Fischer Scientific. RNA estimations were performed using a TECAN Infinite M1000 PRO®.

Assessment of efficiency of SPAAC reaction

For the enzymatic addition of azido-modified ATPs to the 3'-end of RNA, 160 ng/µL RNA was incubated with 1 mM 2'-N_3-2',3'-ddATP 24 U/µL yeast poly(A) polymerase for 1 h at 37 °C. Following precipitation, 50 ng/µL azido-modified RNA and 50 µM DBCO-(PEG4)-biotin (Sigma-Aldrich) were incubated for 1 h at 37 °C, and again precipitated. To digest the RNA to single nucleotides, 25 ng/µL RNA was incubated with 5 mU/µL nuclease P1 for 1 h at 50 °C in 1x P1 digestion buffer. Subsequently, the nucleotides were dephosphorylated by adding FastAP with a final concentration of 50 mU/µL and incubating for 1 h at 37 °C. The reaction was stopped by adding 10% v/v HClO4 (1 M). The nucleosides were analyzed by LC-QQQ (Agilent Ultivo).

Cell culture and confocal imaging

HeLa cells (Sigma Aldrich) were cultured in growth media comprising MEM Earle's media (*Merck*) supplemented with L-glutamine (2 mM), non-essential amino acids (1%), penicillin and streptomycin (1%), and fetal calf serum (FCS, 10%) and under standard conditions (5% CO_2, 37°C). One day before transfection, 2×10^5 cells were seeded in media (1 mL) on 15 mm cover slips in a 12-well cell culture plate. Cells were transfected using Metafectene® Pro (3 µL) in MEM Earle's media (47 µL) and *egfp*/*mcherry* mRNAs (1 µg) in MEM Earl's media (50 µL) for 6 h at 37°C in a total volume of 1 mL per chamber. Then, media with transfection reagent was replaced with fresh growth media. After 24 h, cells were washed twice in 1 x PBS, fixed in 4% paraformaldehyde, washed again twice in 1 x PBS, stained with DAPI (10 µg/µL) for 30 s, washed twice with 1 x PBS and once quickly with water before the coverslips were quickly inverted and mounted on Aqua-Poly/Mount-coated microscope glass slides. Images were taken in using a Leica A TCS SP8 confocal laser scanning microscope with a 63x water-immersion objective and the settings tabulated in Table S2.

Cell culture and western blot analysis

For western blot analysis, HeLa cells were cultured and transfected as described in the imaging experiments. Twenty-four hours after transfection, cells were lyzed using the protocol of the manufacturer for CelLytic™ M (*Sigma Aldrich*). To determine the protein concentration of cell lysate, the Bradford assay was applied by diluting cell lysate (1:50) along with BSA calibration standards (0–10 µg/µL). Samples (50 µL) were then incubated (15 min, rt, exclusion of light) with 1× Roti®-Quant (Roth) staining solution (200 µL) and then, the extinction at 595 nm was determined. Proteins were separated via tris-glycine-PAGE (10% PA, 120 V, 1.5 h, rt, 50 µg of protein) and then the proteins were transferred onto a nitrocellulose membrane Roti®-NC (Roth) in semi-dry transfer buffer with 80 mA for 75 min at rt. After ascertaining transfer efficiency using Ponceaus S-staining, the membrane was cut into suitable pieces for subsequent antibody treatment and washed with 1× PBS + 0.01% Tween (PBST). The membrane was blocked in blocking buffer (3% BSA) for 1 h at rt, followed by incubation with the anti-GFP or anti-α-tubulin (loading control) primary antibodies overnight at 4°C. Then the membrane was washed three times with PBST each for 5 min at rt. The membrane pieces were then incubated with HRP-conjugated secondary antibodies for 1 h at rt and washed three times with PBST afterwards. For chemiluminescence detection the EZ-ECL Chemiluminescence detection kit (*Biological Industries*) was used and results were analysed with a Chemo Star Advanced Fluorescence & ECL Imager (*Intas*). Primary antibodies for GFP (B-2) and α-tubulin were purchased from Santa Cruz Biotechnology and Sigma Aldrich. Secondary antibody polyclonal rabbit anti-Mouse/HRP was from Dako Diagnostics.

Zebrafish work

Wildtype zebrafish (*Danio rerio*) of the AB genetic background were used in all experiments. For the experiment presented in Figure 2B, the fish carried a transgene that directs the expression of GFP on the membrane of PGCs (*tg*(*kop*:*egfp-f'-nos3'UTR-cry*:*dsRed*)).[2] The fish were handled according to the regulations of the state of North Rhine-Westphalia, supervised by the veterinarian office of the city of Muenster.

Construction of plasmids, mRNA synthesis and labeling

For PGC-specific expression of cytoplasmic GFP (*gfp-nos*, internal database no. 355) and for marking germ granules with GFP-tagged Vasa protein (*vasa-gfp*, internal database no. 291), previously published constructs were used.[70, 27] To distinguish between labelled injected mRNA and endogenous mRNA, a *nos* 3'UTR-containing construct was designed (STOP *mcherry-nos*, internal database no. D396). This construct contains an *mcherry* coding sequence, which can be detected in the RNAscope procedure using the mcherry probe. In this construct a single base pair-insertion was introduced following the start codon, leading to a stop codon after 24 nucleotides and no mCherry protein translation. This allowed detection of the red SRB signal of the click-labelled mRNA, with no spectral overlap with mCherry protein. For visualising mRNA via the PP7 detection system, a *nos* 3'UTR-containing construct was designed. In this construct 24 PP7-recognition loop sequences were inserted in the 3'UTR (STOP *nanos-nos 24xpPP7*, internal database no. D016*)*. The PP7 coat protein (PCP) was fused to a nuclear localization sequence (NLS) to direct unbound coat protein to the nucleus, and was tagged with a YFP for visualization of the protein (*nls-ha-tdpcp-yfp-glob*, internal database no. C987). BFP-tagged lifeact expressed in the germ cells using the *nos* 3'UTR (*lifeact-tagbfp-nos*, internal database no. E058) labelled actin rich structures in the PGCs with this fluorescent protein.
Capped sense mRNAs were synthesized using the mMessage mMachine kit (Ambion) according to the protocol of the manufacturer. Then 4000 ng of each mRNA was subjected to enzymatic addition of 2'-N_3-2'-dATP as described above. The click reactions were performed with azido-modified mRNA (1000 ng) and DBCO-conjugated SRB (7.5 µM) for 60 min at 37°C followed by isopropanol precipitation overnight and washing with 70% ethanol. Then, the labeled mRNAs were analyzed on a 7.5% denaturing PA gel.

Microinjection into zebrafish embryos

To visualize the distribution of injected mRNAs in figures 3 and 4, 1 nL containing 80 pg or 200 pg of *gfp-nos* (internal database no. 355) or of STOP *mcherry-nos* (internal database no. D396) mRNAs either non-labeled or labelled with SRB were used. To visualize germ granules with GFP-tagged Vasa protein, 1 nL containing 80 pg of *vasa-gfp* (internal database no. 291) mRNA was injected. For detection of *nos* 3' UTR-containing mRNA using the click-labelling approach and the PP7 detection system in figure 5, 1 nl containing 80 pg of STOP *mcherry-nos* mRNA labeled with SRB were co-injected with an equimolar amount of STOP *nanos-nos 24xPP7* mRNA (internal database no. D016). In addition, 8 pg of *nls-ha-tdpcp-yfp-glob* (internal database no. C987) mRNA were co-injected to express the YFP-tagged PCP protein. To visualize PGCs in figure 5, 80 pg of

lifeact-tagBFP-nos (internal database no. E058) were co-injected. mRNAs were injected into the yolk of 1 cell-stage zebrafish embryos using glass capillaries and the PV830 Pneumatic PicoPump microinjector (WPI).

RNAscope assay

The RNAscope in situ hybridization experiments were performed using the RNAscope Multiplex Fluorescent Reagent Kit (ACD Bio) as previously described.[3] RNAscope probes Probe Diluent (ACD Bio 300041), Dr-nanos3-CDS-C3 (ACD Bio 431191-C3) and mCherry-C2 (ACD Bio 431201-C2) were hybridized overnight at 40°C at a 50:1:1 ratio. Amp4 AltB was used as a label probe combination. DAPI solution was used to label all nuclei.

Microscopy and image analysis in zebrafish embryos

Confocal images were acquired on an LSM 710 microscope (Zeiss) equipped with 405, 488, 561 and 633 nm lasers and a 63x water-immersion objective (Zeiss) controlled by the ZEN software (Zeiss, version 2010B SP1, 6.0). Time-lapses were acquired on a Yokogawa CSU-X1 Spinning Disk microscope equipped with a 63x water-immersion objective (Zeiss) and connected to a Piezo stage and a Hamamatsu Orca Flash 4.0 V3 camera. The microscopy setup was controlled by the VisiView software (Visitron, version 4.0.0.14). For the time-lapse movies, images were acquired at 3.5 seconds intervals over a period of 206.5 seconds using an exposure of 200 ms. For each time point 18 different focal planes with a z-distance of 0.5 µm were acquired. Images were processed using the Fiji software (National Institutes of Health, version 2.0.0-rc-43/1.51a). The 509 nm emission channels showing GFP-positive PGCs in figure 2 were processed with a background subtraction and gaussian filter to reduce signal noise. Images were deconvolved with Huygens Professional version 19.04 (Scientific Volume Imaging, The Netherlands, http://svi.nl), using the CMLE algorithm, with a maximum of 40 iterations.

Supplementary figures

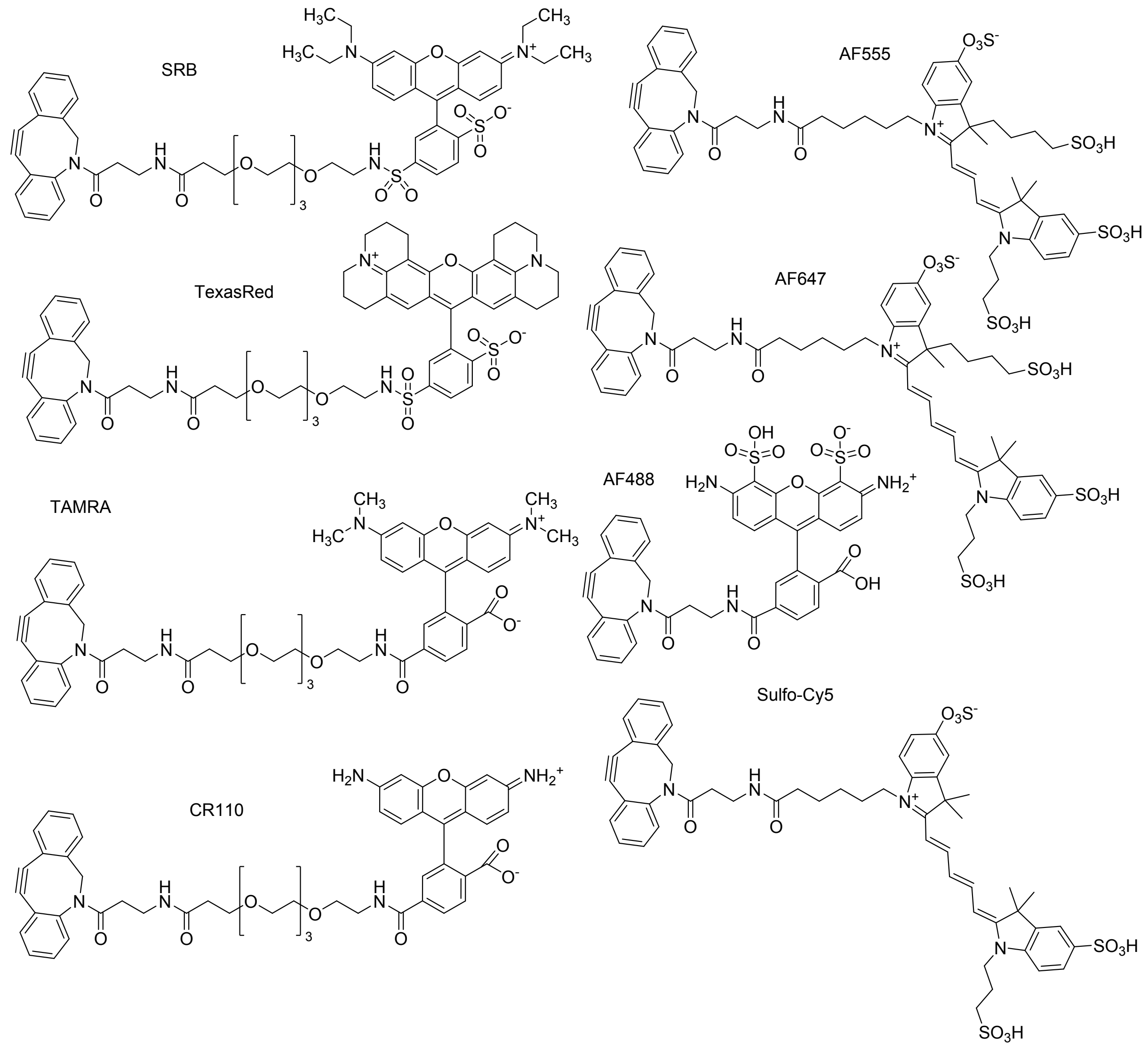

Figure S1. Representative molecular structures of the DBCO-fluorophores used for labeling *egfp* or *mcherry* mRNAs in the study. DBCO-PEG4-5/6-Sulforhodamine B (SRB), DBCO-AF555, DBCO-PEG4-5/6-Texas Red, DBCO-AF647, DBCO-PEG4-5/6-TAMRA, DBCO-AF488, DBCO-PEG4-5/6-Carboxyrhodamine 110 (CR110), DBCO-Sulfo-Cy5 (Jena Biosciences)

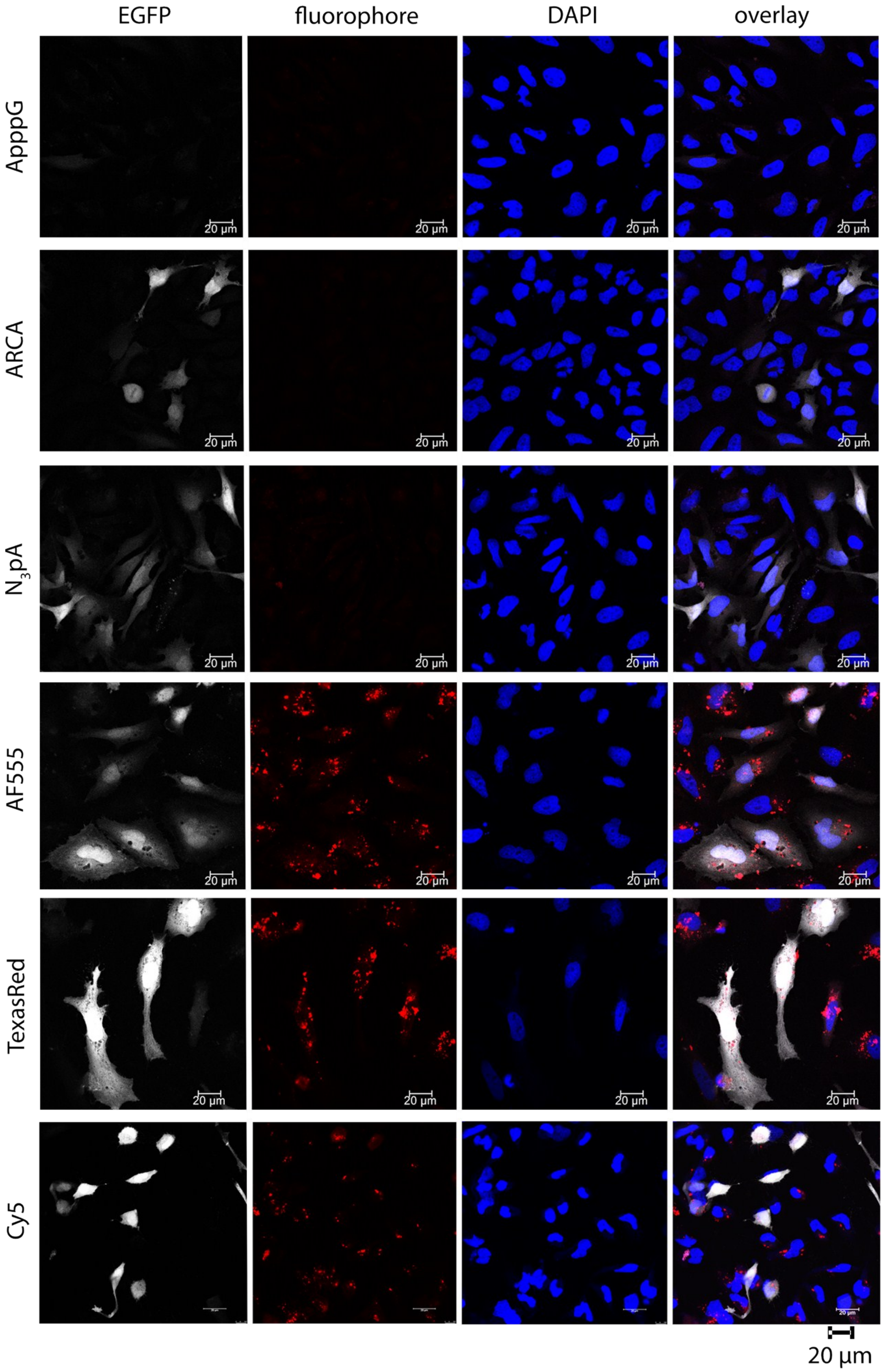

Figure S2. Bioorthogonal labeling of *egfp* mRNAs at the poly(A) tail with different fluorophores enables their visualization in HeLa cells. Confocal microscopy of HeLa cells transfected with *egfp* mRNA unlabeled (ApppG, ARCA, N_3pA) or labeled with the DBCO-containing fluorophores AF555, AF647 or Sulfo-Cy5 (Cy5). Fluorescent signals from the fluorophores (red dots), EGFP and DAPI enabled visualization of mRNA, translated GFP and nuclei, respectively. Scale bar = 20 µm.

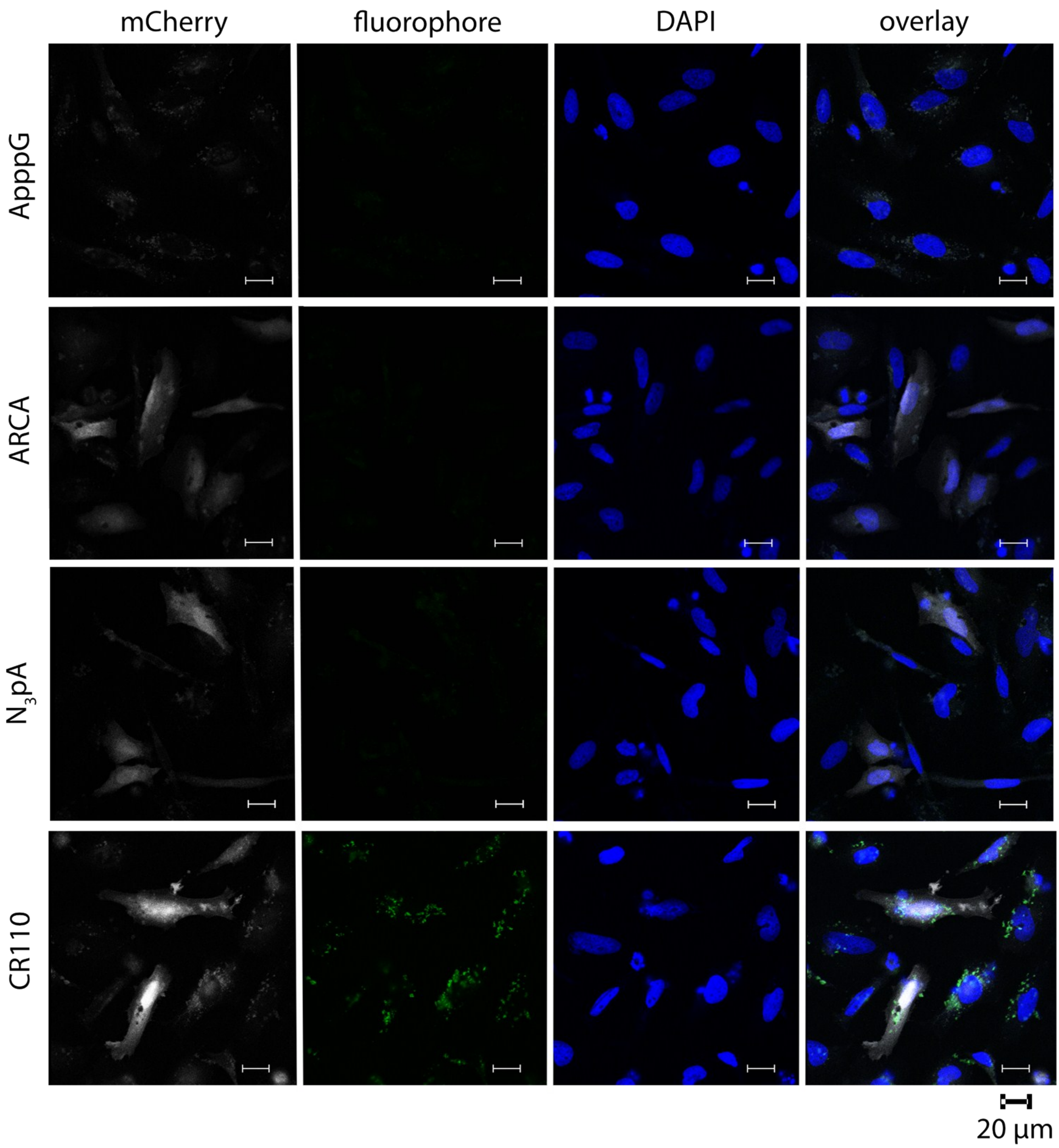

Figure S3. Bioorthogonal labeling of *mcherry* mRNAs at the poly(A) tail with different fluorophores enables their visualization in HeLa cells. Confocal microscopy of HeLa cells transfected with *mcherry* mRNA either unlabeled (ApppG, ARCA, N_3pA) or labeled with the DBCO-containing fluorophores AF488 or carboxyrhodamine 110 (CR110). Fluorescent signals from the fluorophores (green dots), mCherry and DAPI enabled visualization of mRNA, translated mCherry and nuclei, respectively. Scale bar = 20 µm.

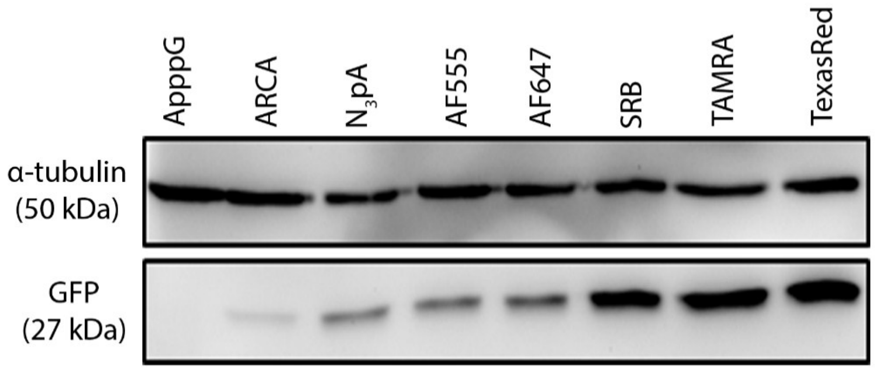

Figure S4. Bioorthogonal labeling of *egfp* mRNAs at the poly(A) tail with different fluorophores does not interfere with their translation. Western blotting showing levels of EGFP in whole-cell lysate of HeLa cells transfected with indicated unlabeled and labeled egfp mRNAs. α-tubulin expression was used as loading control.

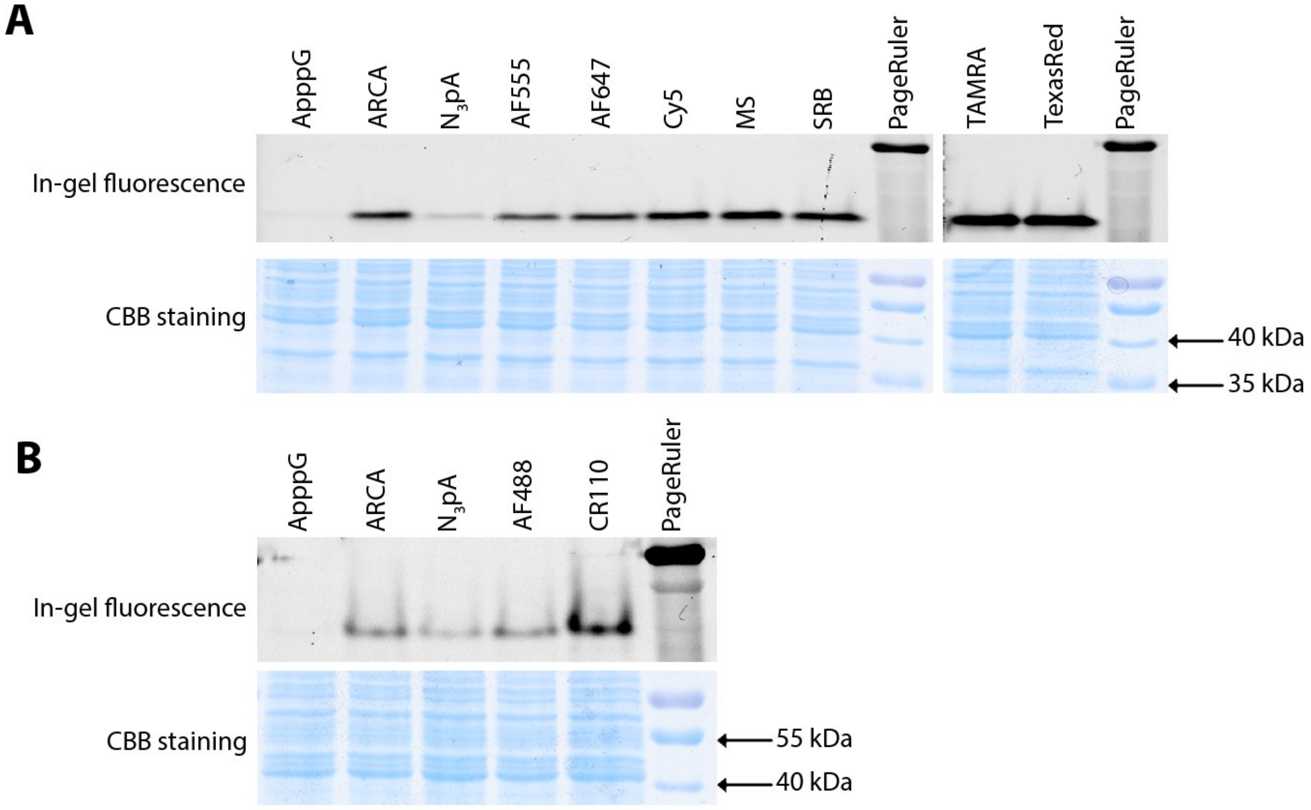

Figure S5. Bioorthogonal labeling of *egfp* mRNAs at the poly(A) tail with different fluorophores does not interfere with their translation. In-gel fluorescence from SDS-PA gels showing levels of EGFP (A) or mCherry (B) (upper panels) and subsequently stained in CBB (lower panels) in whole-cell lysate of HeLa cells transfected with indicated unlabeled and labeled *egfp* or *mcherry* mRNAs.

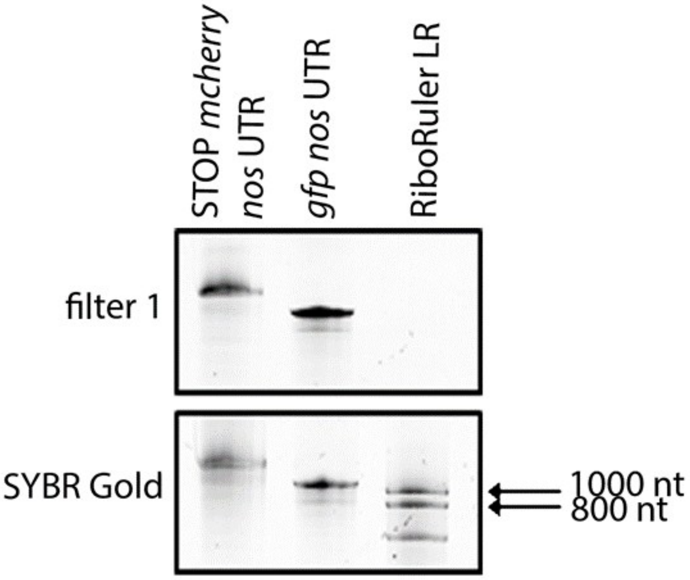

Figure S6. Bioorthogonal labeling of STOP *mcherry nos* UTR and *gfp nos* UTR is efficient with DBCO-sulforhodamine B. The *egfp-nos* UTR and STOP *mcherry-nos* UTR mRNAs clicked with DBCO-SRB. PAGE analysis of *egfp-nos* UTR and STOP *mcherry-nos* UTR mRNAs (7.5% PA gel, 20 W, 1.5 h, rt) labeled with DBCO-SRB. Fluorescent signals from SRB observed using filter 1 (≥ 575 nm) and SYBR^{TM} Gold staining are shown.

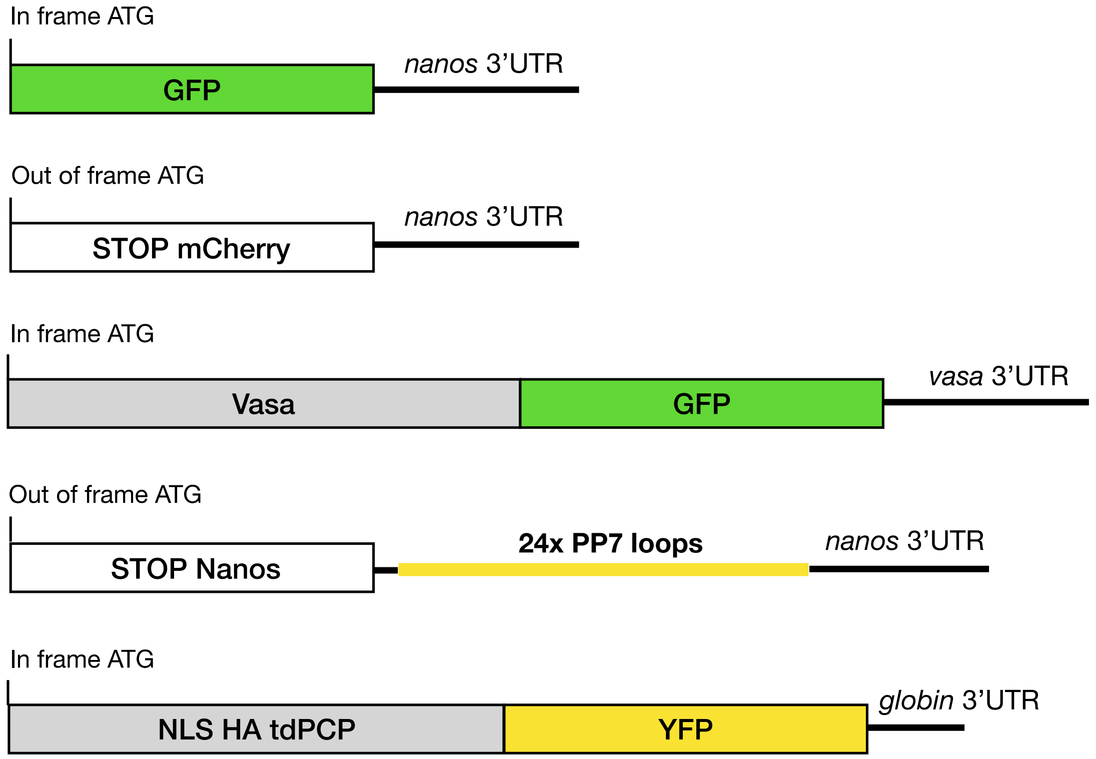

Figure S7. Scheme of RNA constructs used for labeling and injection. The STOP mcherry *nanos* 3'UTR (STOP mCherry nos) construct is not translatable due to a frame shift in the coding sequence shortly after the initiation codon. Likewise, the STOP Nanos 24xPP7 construct is not translatable due to a mutation in the ATG start codon. GFP = 714 bp, STOP mCherry = 709 bp, *nanos* 3'UTR = 631 bp, Vasa = 1660 bp, *vasa* 3'UTR = 498 bp, STOP Nanos = 479 bp, PP7 loop sequence = 1450 bp, NLS HA tdPCP = 1602 bp, *globin* 3'UTR = 191 bp.

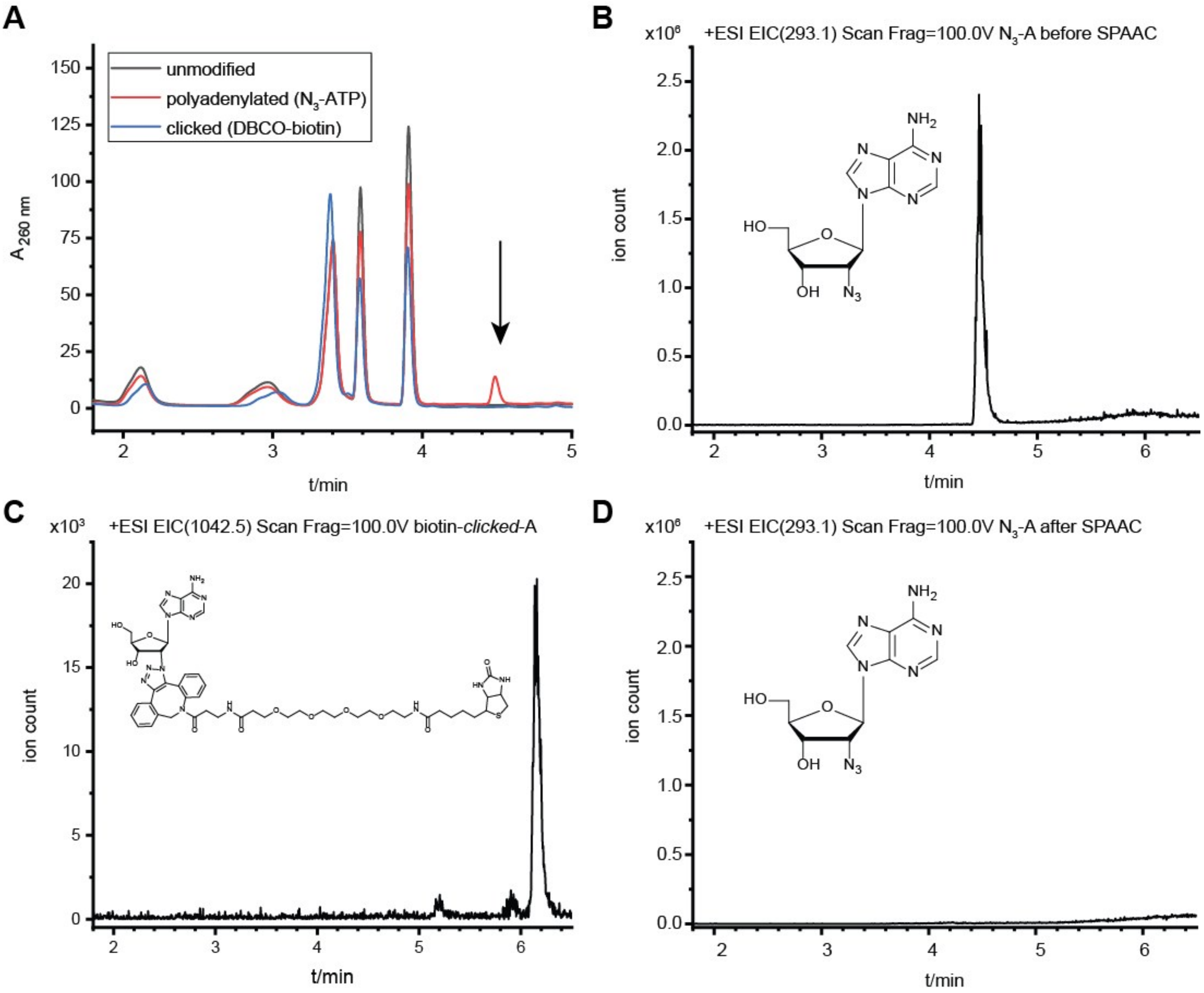

Figure S8 HPLC and LC-QQQ analysis of SPAAC reaction of RNAs modified at the 3′ end with 2′-azido-ddATP. RNAs were modified as indicated, then digested and dephosphorylated to single nucleosides for analysis. A) HPLC analysis at 260 nm shows the four nucleosides and azido-A (arrow) in the case of enzymatic modifications (red trace). After the click reaction, no azido-A is detectable, indicating complete conversion (blue trace), similar to unmodified RNA (black trace). B) EIC for azido-A (M+H = 293.10), confirming the mass of the peak. C) EIC of the mass expected for the click product of azido-A with biotin (M+H= 1042.45), showing that the expected product is formed. D) EIC for azido-A after the click reaction showing that no azido-A can be detected after the click reaction and also confirming complete conversion during the SPAAC reaction.

Supplementary tables

Table S1. Settings for measurement of fluorescent signals on the Typhoon FLA 9500 scanner

Fluorophore	λ_{max} [nm]	λ_{em} [nm]	excitation laser [nm]	scanning filter
SYBR Gold	495	537	473	LPB
AF488	494	517	473	LPB
CR110	501	526	473	LPB
AF555	555	572	532	LPG
TAMRA	560	565	532	LPG
SRB	568	585	532	LPG
TexasRed	586	601	532	LPG
MegaStokes	486	608	532	LPG
Sulfo-Cy5	646	661	635	LPR
AF647	648	671	635	LPR
mCherry	587	610	532	LPB
EGFP	488	510	473	LPB

Table S2. Settings for detection of fluorescent signals on the Leica A TCS SP8 confocal laser scanning microscope

Fluorescent signal	λ_{max} [nm]	λ_{em} [nm]	excitation laser [nm]	emission filter
DAPI	358	461	405	LP 425
AF488	494	517	488	BP 527/30
CR110	501	526	488	BP 527/30
EGFP	488	510	488	BP 527/30
AF555	555	572	552	LP 590
TAMRA	560	565	552	LP 590
SRB	568	585	552	LP 590
TexasRed	586	601	552	LP 590
mCherry	587	610	552	BP 700/75
Sulfo-Cy5	646	661	638	BP 700/75
AF647	648	671	638	BP 700/75

References

[1] J. M. Holstein, L. Anhäuser, A. Rentmeister, *Angewandte Chemie - International Edition* **2016**, *55*, 10899-10903.

[2] H. Blaser, S. Eisenbeiss, M. Neumann, M. Reichman-Fried, B. Thisse, C. Thisse, E. Raz, *Journal of Cell Science* **2005**, *118*, 4027-4038.

[3] T. Gross-Thebing, A. Paksa, E. Raz, *BMC Biology* **2014**.

Author Contributions

K.W. and E.R. designed and K.W. performed the zebrafish-related experiments (Figures 3, 4, 5, Supplementary Figure 7 and supplemental movie 1) and synthesized capped sense mRNAs used for bioorthogonal labeling in these figures. K.W. and E.R. wrote the parts of the manuscript relevant for Figures 3, 4, 5, supplementary figure 7 and supplemental movie 1). T.G-T. established and tested the PP7 RNA detection system and contributed plasmids used in Figures 3, 4 and 5. K.S.C. and A.R. designed the experiments pertaining to mRNA labeling and cell culture experiments. K.S.C. performed the experiments presented in Figure 2, Supplemental figures 2-6, and labeled the mRNAs used in experiments presented in Figures 3 and 4, and supplemental movie S1. N.K. labeled the mRNAs used in Figure 5 and performed experiments presented in Figure S8. K.S.C. and A.R. wrote the parts of the manuscript relevant to these figures and the introduction. E.R. and A.R. supervised the biology/zebrafish and chemistry/cell culture parts of the project, respectively. All authors read and approved the final manuscript.
